# Enhanced Extinction of Aversive Memories by High-Frequency Stimulation of the Rat Infralimbic Cortex

**DOI:** 10.1371/journal.pone.0035853

**Published:** 2012-05-07

**Authors:** Mouna Maroun, Alexandra Kavushansky, Andrew Holmes, Cara Wellman, Helen Motanis

**Affiliations:** 1 Department of Neurobiology, Faculty of Natural Sciences, University of Haifa, Haifa, Israel; 2 Laboratory of Behavioral and Genomic Neuroscience, National Institute on Alcoholism and Alcohol Abuse, National Institutes of Health, Bethesda, Maryland, United States of America; 3 Department of Psychological and Brain Sciences and Center for the Integrative Study of Animal Behavior, Indiana University, Bloomington, Indiana, United States of America; Utrecht University, The Netherlands

## Abstract

Electrical stimulation of the rodent medial prefrontal cortex (mPFC), including the infralimbic cortex (IL), immediately prior to or during fear extinction training facilitates extinction memory. Here we examined the effects of high-frequency stimulation (HFS) of the rat IL either prior to conditioning or following retrieval of the conditioned memory, on extinction of Pavlovian fear and conditioned taste aversion (CTA). IL-HFS applied immediately after fear memory retrieval, but not three hours after retrieval or prior to conditioning, subsequently reduced freezing during fear extinction. Similarly, IL-HFS given immediately, but not three hours after, retrieval of a CTA memory reduced aversion during extinction. These data indicate that HFS of the IL may be an effective method for reducing both learned fear and learned aversion.

## Introduction

Experimental extinction is the decline in the frequency or intensity of a conditioned response following the withdrawal of reinforcement, and is believed to reflect relearning rather than unlearning [1]–[4]. A reduced ability to extinguish conditioned fear associations might contribute to the persistence of maladaptive fear in conditions such as posttraumatic stress disorder (PTSD) and may reduce the effectiveness of therapeutic interventions that rely on extinction processes [5]–[8].

The infralimbic subregion (IL) of medial prefrontal cortex (mPFC) has been suggested to play a key role in extinction of aversive associations as measured using Pavlovian conditioned fear and taste aversion paradigms [9]–[16]. For example, various pharmacological and electrophysiological manipulations within the IL modify the ability to extinguish aversive memories [9]–[10], [14], [16]–[18].

Electrical stimulation of the IL during extinction training in a manner that mimics conditioned stimulus-induced firing has been found to reduce the expression of conditioned fear and enhance learning and/or expression of extinction in rats [9], [18]–[19]. In addition, high-frequency stimulation (HFS) of the mediodorsal thalamic inputs to the mPFC, including IL, immediately prior to extinction learning facilitates extinction memory in mice, whereas low-frequency stimulation has the opposite effect [20]–[21]. Extinction is also associated with plasticity changes in inputs from, as well as outputs to, the basolateral amygdala (BLA), in the form of augmentation of evoked field potentials (EFPs) in the BLA-mPFC pathway and depression of EFPs in the reciprocal mPFC-BLA pathway [22].

These studies raise the possibility that environmental events that are risk factors for neuropsychiatric disorders such as PTSD might act in part by modifying mPFC plasticity. Interestingly in this context, exposure to stress both reduces HFS-induced potentiation of plasticity in the mPFC [23]–[24] and impairs fear extinction ]25–29]. Preliminary clinical evidence indicates that successful exposure therapy in PTSD is associated with a shift from depression to potentiation of mPFC neuronal activity [30], suggesting that the findings in rodent models could have clinical implications. As such, it is important to further characterize the nature and generalizability of the link between mPFC plasticity and extinction.

The major aims of the current study were twofold: (1) to examine the effects of the application of HFS to the IL on the consolidation phase of extinction, and (2) to determine whether the effects of IL-HFS on extinction were specific to fear, or also affected another form of IL-mediated [11], [31] extinction, conditioned taste aversion (CTA).

## Materials and Methods

### Animals and Surgery

Subjects were male Wistar rats (∼60 days old, 250–300 g) purchased from Harlan, Israel. Upon arrival animals were housed 5 per cage in a 22±2°C vivarium under a 12-h light/dark cycle. Water and food were available *ad libitum* throughout the experiment unless otherwise indicated. All experiments were approved by the University of Haifa Ethics and Animal Care Committee, and conducted in accordance with NIH guidelines for minimizing pain and discomfort. A week after arrival, the rats were anaesthetized with 4.8 ml/kg Equithesin (2.12% w/v MgSO_4_, 10% v/v ethanol, 39.1% v/v propylene glycol, 0.98% w/v sodium pentobarbital, and 4.2% w/v chloral hydrate), and placed in a stereotaxic frame, with body temperature maintained at 37±0.5°C. Twisted stimulating electrodes targeting IL (anteroposterior: +3 mm relative to bregma, lateral: ±0.5 mm; ventral: 4–5 mm) were bilaterally implanted and affixed with dental cement. Following the surgery, animals were housed individually and left undisturbed for one week to recuperate.

### Fear Conditioning, Extinction and Reconditioning

One week following the surgery, animals were habituated for three days to transportation, to context B, a chamber with transparent Plexiglas walls and black Plexiglas floor in which extinction training took place (via 20 min exposure), to the test room, and to being connected to a head-stage commutator.

Fear conditioning was conducted in context A, a chamber with a grid floor and transparent Plexiglas walls. The conditioning procedure was as previously described [32] and comprised 3x pairings of a conditioned stimulus (CS) and unconditioned stimulus (US) (120-sec inter-pairing interval) after a 120-sec no-stimulus baseline. The CS was a 4 kHz, 80 dB 30-sec tone that co-terminated with delivery of the 0.8 mA, 1-sec footshock US. The day after conditioning, rats were placed in context B (different from context A in shape and size) and given a fear retrieval/short extinction session entailing 5xCS. Full extinction was carried out in context B on the next 2 days by presentation of 10xCS.

Freezing, the absence of all movement except for respiration [33]–[34], was quantified from video by image-based software (P. Schmid, Behavioral Neurobiology Laboratory, Swiss Federal Institute of Technology Zurich). Extinction results are presented as the percent time spent freezing during the 30 sec tone. The results for the 10 tones in each of the extinction trainings are presented as 5 sessions, each consisting of the average of 2 trials. For the retrieval test, freezing to the 1st and the 5^th^ tones is presented.

For reconditioning, 1 day after extinction training, rats were placed in context A and given a single CS-US pairing. Beginning the next day, rats were given daily extinction sessions over 2 days (as above).

### Conditioned Taste Aversion (CTA) Acquisition, Extinction and Reconditioning

Rats underwent surgery, electrode implantation and recovery as above. CTA was conducted as previously described [3], [26], [35]–[37]. Rats were water deprived for 23.5 hr/day and then, over 3 consecutive days, trained to drink water from 2 pipettes each containing 10 mL of tap water during daily 20 min access sessions. During these days, animals were habituated to the stimulation chamber for 20 min a day and for 3 days.

On the conditioning day, water was replaced with saccharin (0.1% w/v) during the 20 min access session. Twenty min later, rats received intraperitoneal injections of lithium chloride (LiCl, 0.15 M, 2% body weight) to induce malaise. Three days after conditioning [3], [11], [16] rats were given a fear retrieval/extinction session entailing 10-min access to saccharin followed by 10-min access to water to prevent dehydration. Daily extinction sessions were conducted over the next 2 days. These entailed 10-min access to saccharin, followed by 10-min access to water to prevent dehydration.

There were then 2 test-free days during which rats were given 20-min daily water access sessions to prevent dehydration. Reconditioning occurred the next day. Rats were given 20-min access to a NaCl solution (0.3%) and, 20 min later, injected with LiCl to induce malaise (as above). After a 2-day interval another set of daily extinction sessions were conducted over the next 2 days, involving 10-min access to NaCl, followed by 10-min access to water. All behavioral measurements of CTA were carried out in the home cage.

CTA was measured as an aversion index, defined as mL of water drunk/total fluid (water+tastant drunk×100, with a score of 100 indicating complete CTA and a score of 50 indicating no CTA and no preference for the tastant.

### IL High-Frequency Stimulation (HFS)

The HFS protocol was conducted as previously described [23], [38]–[39]. Stimulation was given in trains of 10×100 Hz (TS-100) pulses, with 10 trains applied in a row (200 msec inter-train interval). There were 3 sets of 10x trains in total (120 sec inter-set interval). The stimulation procedure lasted 10 min. Non-stimulated controls were connected to the head-stage for the equivalent period but received no stimulation.

**Figure 1:**
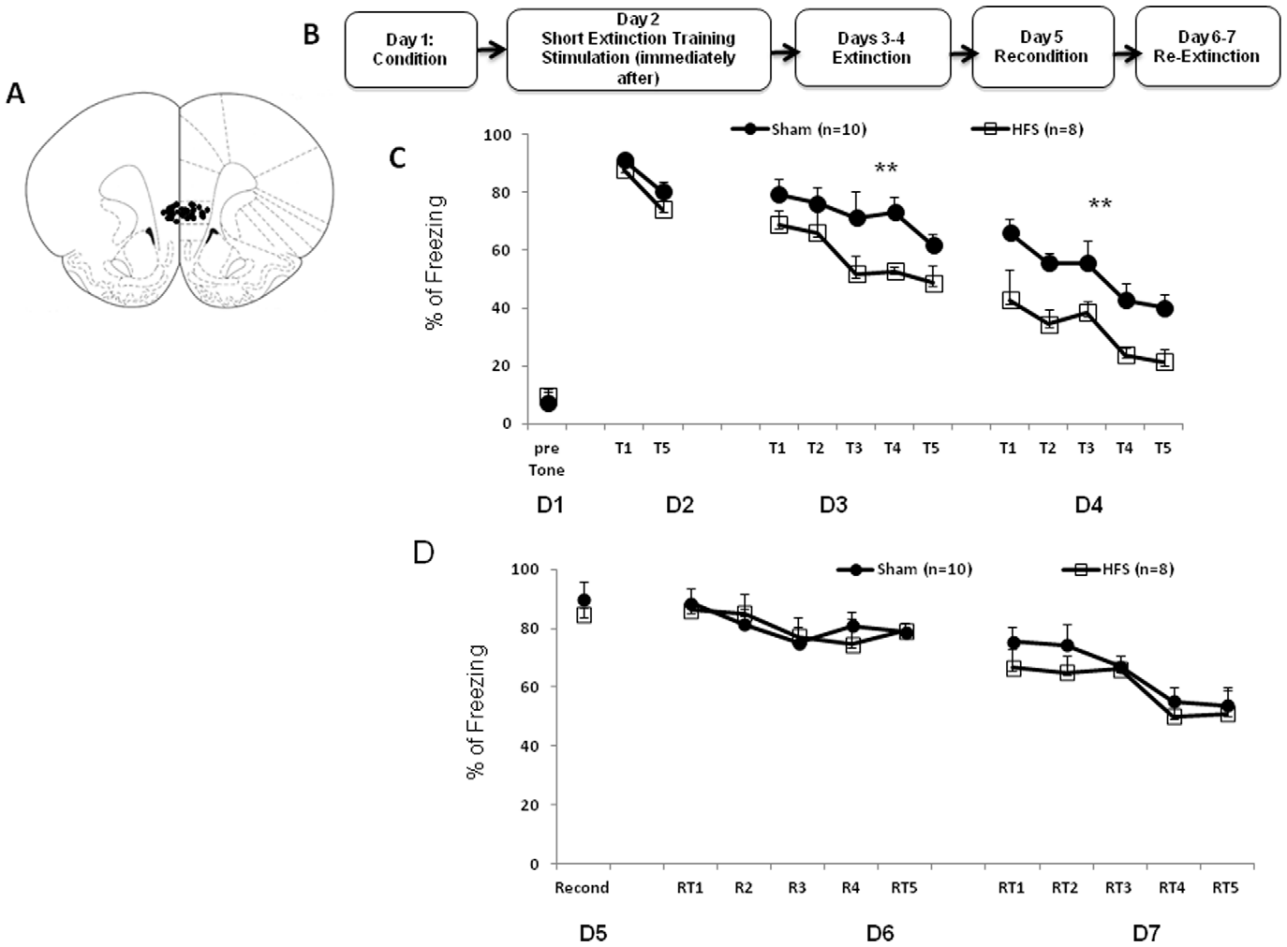
IL-HFS applied immediately after fear retrieval/short extinction training reduces fear during extinction. (**A**) Schematic diagram showing electrode placement in IL for the fear conditioning experiments. Diagram adapted from Paxinos and Watson 1998. (**B**) Timeline of behavioral testing and stimulation. (**C**) Percent freezing (mean ± SEM) to CS during conditioning, fear retrieval, and extinction in rats receiving either sham or HFS after fear retrieval. HFS rats showed reduced fear during extinction relative to Shams (**P<.01). (**D**) Percent freezing to CS during reconditioning and extinction in rats receiving either sham or HFS after fear retrieval. Reconditioning and re-extinction were not affected by prior IL stimulation. Data are Means ±SEM.

**Figure 2:**
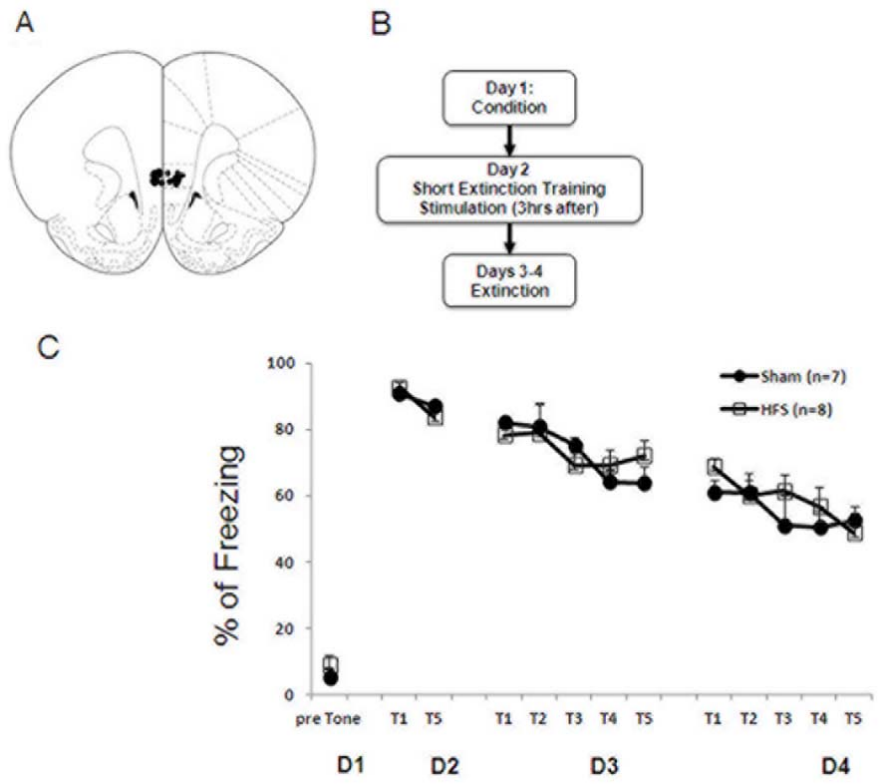
IL-HFS applied three hours after fear retrieval/short extinction training does not alter fear during extinction. (**A**) Schematic diagram showing electrode placement in IL [56] for the fear conditioning experiments. (**B**) Timeline of behavioral testing and stimulation. (**C**) Percent freezing to CS during conditioning, fear retrieval, and extinction in rats receiving either sham or HFS prior to conditioning. Sham and HFS rats did not differ during any phase. Data are Means ±SEM.

**Figure 3:**
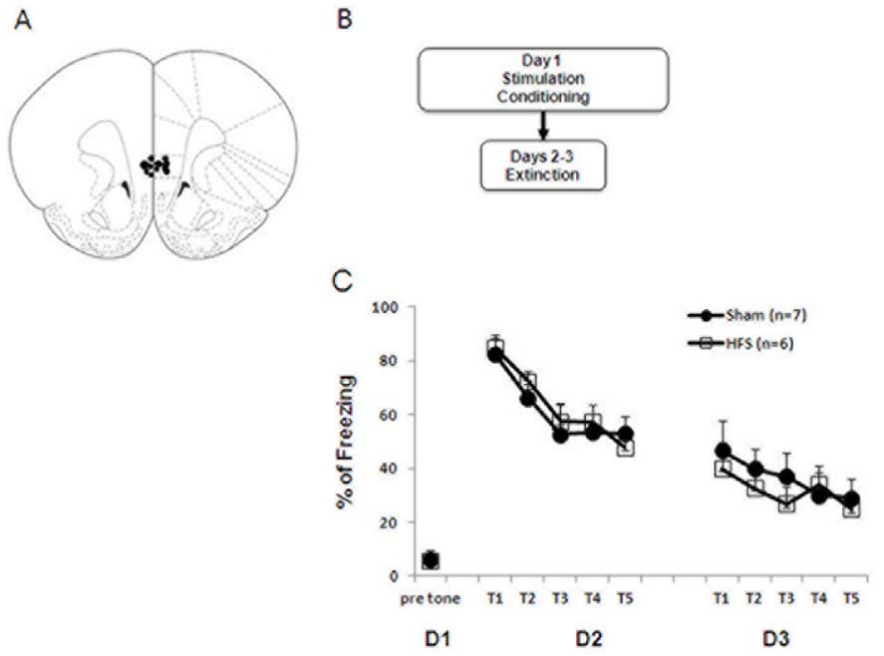
IL-HFS applied immediately before conditioning does not alter fear during extinction. (**A**) Schematic diagram showing electrode placement in IL [56] for the fear conditioning experiments. (**B**) Timeline of behavioral testing and stimulation. (**C**) Percent freezing to CS during conditioning, fear retrieval, and extinction in rats receiving either sham or HFS prior to conditioning. Sham and HFS rats did not differ during any phase. Data are Means ±SEM.

*IL-HFS on fear extinction* In 3 separate experiments, HFS was applied either (1) immediately following fear retrieval and the short extinction protocol (**Figure 1B**), (2) 3 hrs after fear retrieval and the short extinction protocol (**Figure 2A**) or (3) before fear conditioning (**Figure 3A**). In each case, rats were placed in the stimulation chamber and given either HFS or no stimulation.

#### *IL-HFS* on conditioned taste aversion

**Figure 4:**
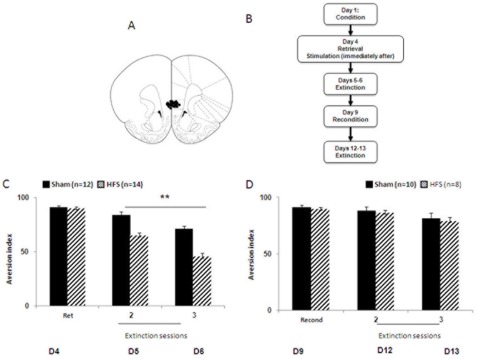
IL-HFS applied immediately after CTA retrieval/short extinction reduces aversion index during extinction. (**A**) Schematic diagram showing electrode placement in IL [56] for the CTA experiment. (**B**) Timeline of behavioral testing and stimulation. (**C**) Aversion index (mL water drunk/total fluid drunk × 100) during conditioning, retrieval, and extinction in rats receiving either sham or HFS after retrieval. HFS rats showed reduced aversion during extinction relative to Shams. **P<.01. (**D**) Aversion index during CTA to a new tastant and extinction in rats receiving either sham or HFS after retrieval. Conditioning to the new tastant and re-extinction were not affected by prior IL stimulation. Data are Means ±SEM.

**Figure 5:**
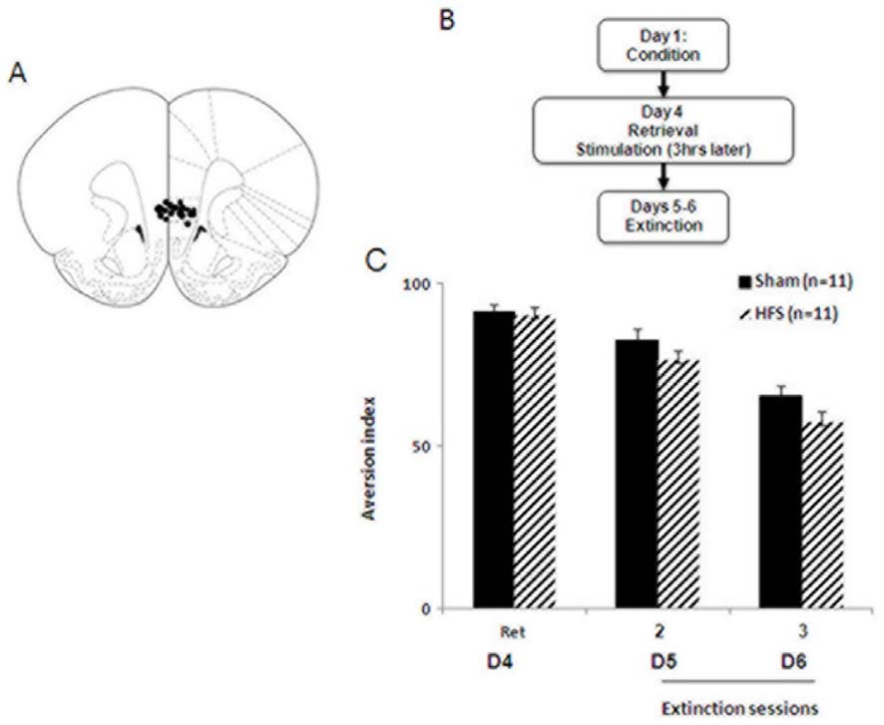
IL-HFS applied immediately after CTA retrieval/short extinction does not affect the aversion index during extinction. (**A**) Schematic diagram showing electrode placement in IL for the CTA experiment. (**B**) Timeline of behavioral testing and stimulation. (**C**) Aversion index (mL water drunk/total fluid drunk×100) during conditioning, retrieval, and extinction in rats receiving either sham or HFS 3 hrs after retrieval. HFS rats were not different from Shams. Data are Means ±SEM.

In 2 separate experiments, HFS was applied either (1) immediately following CTA retrieval (**Figure 4B**) or (2) following fear retrieval (**Figure 5A**).

### Histology

After the last session of behavioral testing, rats were deeply anaesthetized with an overdose of Equithesin and marking lesions were made by passing anodal currents (10 mA for 3 sec) through the metal electrodes. Electrode tips were examined under a light microscope following Nissl staining. **Figures 1A** **and** **4A** for the fear conditioning and CTA experiments, respectively, show a schematic drawing of the mPFC (coronal view at position +3.20 and +2.70 mm anterior to bregma). Solid black circles indicate the locations.

### Statistics

Differences between groups and across the testing days were determined using Student’s t-tests or mixed-design analysis of variance (ANOVA) followed by LSD *post hoc* tests.

## Results

### IL-HFS Immediately After Fear Retrieval Enhances Fear Extinction

Prior to stimulation, the two stimulation groups did not differ in freezing during the fear memory retrieval test (t-test: *p*>.05).

Effects of stimulation group and training-block on freezing during extinction were analyzed using a 2-factor ANOVA, with repeated measures for training block. There were significant effects of stimulation group (F1,16 = 7.2 *p*<.05) and training block (F1,16 = 58.2, *p*<.01), but no interaction (*p*>.05). Freezing to the CS was lesser in stimulated than non-stimulated rats, and both groups showed significant reductions in freezing across training blocks (**Figure 1C**). This demonstrates reduced fear during extinction as a result of IL stimulation after the short extinction training. To determine more precisely when the facilitation of extinction emerged, freezing was compared for each of the 5 blocks on the first day of extinction. The results show that the groups did not differ at either T1 or T2 (t-test: *p*>.05) and the facilitation of extinction emerged during the T3 block (t(16) = 2.2; *p*<.05). These suggest that IL stimulation did not affect the expression of conditioned fear but facilitated acquisition of extinction.

We next tested whether the effects of IL stimulation would persist after rats had been reconditioned to the original fear memory. Twenty-four hrs after extinction training, rats were placed in context A and given a single CS-US pairing. Beginning the next day, rats were given daily extinction sessions over 2 days (as above).

Results showed that groups did not differ in freezing to the CS during the reconditioning session (t-test: *p*>.05) or any of the extinction training-blocks (2-factor ANOVA main effect and interaction: *p*>.05), although there was a significant decrease in freezing with training-block (F1,16 = 16.4, *p*<.01, **Figure 1D**). This shows that the effect of earlier IL stimulation was occluded by reconditioning. The fact that the stimulated group showed normal fear extinction after reconditioning also discounts any stimulation-induced mechanical damage to IL.

### IL-HFS 3 Hours After Fear Retrieval does not Enhance Fear Extinction

To determine the upper limit of the time window of the effects of IL-HFS, animals received HFS three hrs following fear retrieval. A previous work showed that the microinfusion of anisomycin, the protein synthesis inhibitor, into the mPFC three hrs post retrieval did not affect reconsolidation of object recognition [40]. On extinction prior to stimulation, the two stimulation groups did not differ in freezing during the fear memory retrieval test (t-test: *p*>.05).

Effects of stimulation group and training block on freezing during extinction were analyzed using a 2-factor ANOVA, with repeated measures for training block. There was a significant effect of training block (F1,13 = 18.9, *p*<.01) but not stimulation group and no interaction (*p*>.05). Both groups showed significant reductions in freezing across training blocks (**Figure 2C**). These results suggest a critical post-retrieval time window for HFS to affect extinction.

### IL-HFS Prior to Fear Conditioning does not Alter Fear Retrieval or Fear Extinction

During conditioning, groups did not differ in freezing to the CS during the first trial (i.e., prior to pairing with the US) (t-test: *p*>.05), indicating no effect of HFS on unconditioned fear (**Figure 3C**). Freezing to the CS on trial 3 was high (∼80%) and similar between the groups (t-test: *p*>.05), demonstrating no difference in fear acquisition. A 2-factor ANOVA was used to analyze the effects of stimulation group and training block (repeated measures for training block) on freezing during extinction. There was a significant effect of training block on freezing (F1,11 = 5.2, *p*<.05, see **Figure 3C**), but not of group and no interaction (ANOVA: *p*>.05). These data demonstrate that IL stimulation prior to conditioning did not affect fear acquisition or alter the manner in which fear was learned such that later fear extinction was affected. This is consistent with the view that IL has a minimal role in fear learning [12]–[13], [28].

### IL-HFS Immediately After Fear Retrieval Enhances CTA Extinction

Stimulation groups did not differ in saccharin consumption either prior to LiCl injection (Sham: 13.8±0.8 ml; HFS: 12.9±0.4 ml, t-test: *p*>.05) or during the retrieval test prior to stimulation (t-test: *p*>.05, **Figure 4C**). Effects of stimulation group and extinction-session on the aversion index were analyzed using a 2-factor ANOVA, with repeated measures for extinction-session. There were significant effects of stimulation (F1,24 = 41.1, *p*<.01) and extinction session (F1,24 = 49.84, *p*<.01), but no interaction (*p*>.05). The aversion index was less in stimulated than non-stimulated rats, and both groups showed significant reductions in the index across training-blocks (**Figure 4C**). This demonstrates reduced aversion during extinction as a result of post-retrieval IL stimulation, similar to the effects on fear extinction.

We next tested whether the effects of IL stimulation would persist after rats were given CTA conditioning to a novel tastant. During the 2 days after extinction, rats were given 20-min daily water access sessions to prevent dehydration. The next days, rats were given 20-min access to a NaCl solution (0.3%) and, 20 min later, injected with LiCl to induce malaise (as above). Daily extinction sessions were conducted over the next 2 days, involving 10-min access to water, followed by 10-min access to NaCl.

Results showed that the aversion index did not differ between groups during the new conditioning session (t-test: *p*>.05) or extinction sessions (2-factor ANOVA main effect and interaction: *p*>.05), although there was no significant decrease in aversion across extinction session after this second conditioning (**Figure 4D**). This shows that the effects of earlier IL stimulation were absent after new conditioning, similar to the lack of lasting effects on fear reconditioning.

### IL-HFS 3 Hours After Fear Retrieval does not Enhance CTA Extinction

Stimulation groups did not differ in their aversion before the application of HFS (t-test: *p>*0.05). A 2-factor ANOVA was used to analyze the effects of stimulation group and training block (repeated measures for training block) on the aversion index during extinction. There was a significant effects of extinction session (F1,20 = 102.5, *p*<.01 ) but not HFS and no interaction (*p*>.05) for aversion scores during extinction. Both groups showed significant reductions in the index across training-blocks (**Figure 5**C). These results suggest that HFS has no effect on extinction of CTA when applied 3 hrs after the retrieval of CTA.

## Discussion

The main finding of the current study was that HFS of the IL following retrieval of a conditioned fear memory led to significant reductions in fear during extinction. Another novel finding was that the same effect of IL-HFS was produced in a CTA paradigm. In both paradigms, IL-HFS applied three hours after retrieval did not affect extinction.

Previous studies have shown that microstimulation of IL immediately (i.e., 100–400 msec) after CS presentation decreases the expression of conditioned fear during extinction training and enhances long-term extinction memory [9], [19]. Current findings extend these data by demonstrating that HFS of the IL after a 5xCS fear retrieval session is sufficient to reduce fear, and CTA, during extinction training beginning one day after stimulation. Thus, IL stimulation produced significant and lasting reductions in learned fear and aversions even without tight temporal coupling of the CS and the stimulation. These effects were specific to immediate post-retrieval HFS, as the same stimulation protocol applied either three hours after retrieval or prior to conditioning did not affect fear acquisition or fear during subsequent extinction. The lack of changes in fear acquisition is consistent with the inability of IL/mPFC lesions to alter fear learning or expression [12]–[13], [28], while the absence of effects of delayed stimulation indicates a critical temporal window for HFS application. This latter finding is consistent with previous work showing that the microinfusion of anisomycin, the protein synthesis inhibitor, into the mPFC three hrs post retrieval did not affect reconsolidation of object recognition [40].

One possible account of the observed pattern of effects is that HFS applied immediately after retrieval disrupted reconsolidation of the original fear/CTA memory, similar to the effects of protein synthesis inhibitors [41]–[45], leading to partial erasure of the original memory. However, this is perhaps a less parsimonious explanation than a decrease in fear/CTA produced by a strengthening of an IL-mediated inhibitory fear memory, for a number of reasons. First are the aforementioned studies demonstrating that IL stimulation facilitates extinction, as well as the wider literature on IL-mediated suppression of conditioned fear via connections to the amygdala (see below). Second, the original fear memory was very readily reinstated in a manner that suggested weakening of an inhibitory memory (following HFS facilitation of extinction) rather than generation of a fear memory de novo (after erasure of an old memory via disruption of reconsolidation). Third, the relatively long retrieval procedure employed in the fear conditioning experiments, comprised of five CS presentations, likely favors extinction over reactivation, suggesting that post-retrieval HFS may have facilitated consolidation of a partial extinction memory. Indeed, microinfusion of a protein synthesis inhibitor into IL disrupts consolidation of both fear extinction [14] and CTA extinction following the same behavioral protocol [11].

It should be noted, however, that the kinetics of extinction following reconditioning were different in the CTA as compared to the fear conditioning paradigm. Notably, while in the fear conditioning paradigm, animals readily show a good ability to re-extinguish, in the CTA paradigm both sham and HFS groups seem to have resistance to extinction following reconditioning. It was previously shown that extinction is slower following double training in CTA [3], [36]. Regardless, in both paradigms the return of fear suggests that the original memory was not abolished.

Notwithstanding these issues, an effect of HFS on reconsolidation cannot be fully discounted from the current data. Indeed, this would be a worthy direction in and of itself given recent interest in the therapeutic potential of manipulations affecting reconsolidation [46]–[47]. In fact, if indeed the effects we observed do occur through disruption of reconsolidation, then they would be the first demonstration that IL stimulation can bolster this effect. This suggests that appropriately timed stimulation of an input to a structure in which reconsolidation is occurring can also disrupt reconsolidation.

An important avenue for future work will be elucidating the mechanisms underlying HFS-induced reductions in fear. In this context, HFS has been found to induce NMDA receptor-mediated long-term potentiation (LTP) in the mPFC [23], [48], and we recently found that fear extinction is accompanied by potentiation and LTP-like excitatory plasticity in mPFC [22]. Additionally, fear extinction has been shown to be dependent upon activation of mPFC NMDA receptors [17] and correlates with NMDA-mediated mPFC neuronal bursting [49]. Together, these findings suggest that the induction of NMDA-mediated LTP could be a substrate for the fear inhibitory effects of IL-HFS, which results in facilitation of extinction. Extinction consolidation is associated with strong activation of c-Fos in the mPFC [31], [50] and resistance to extinction is associated with impaired IEG induction in the mPFC [50]. Given the role of NMDA receptors in controlling immediate early gene (IEG) transcription, these findings suggest that activation of NMDA-mediated processes may be the primary cause of such IEG activation [50]. Furthermore, and in support of the link between changes in plasticity in the IL and facilitation of extinction, it was previously shown that induction of long-term depression in the mPFC is predictive of spontaneous recovery of conditioned fear and resistance to extinction [20]–[21] and these are associated with failure in IEG induction in the mPFC [50].

A corollary question is how HFS-driven plasticity changes in IL modify the broader corticolimbic circuit mediating fear and extinction. The IL sends projections to the basolateral amygdala, as well as intercalated cell masses that provide feedforward inhibition of the central amygdala [51]–[54]. This latter projection has been posited to be a pathway for the IL to inhibit fear responses by suppressing amygdala output [55], and provides a plausible mechanism by which HFS potentiation of IL activity could reduce fear. We propose that the facilitation of extinction is produced following the application of HFS to the IL which seemingly induces an NMDA-dependent potentiation that results in a powerful inhibition of the amygdala by the IL.

In summary, the current study provides novel evidence that HFS of the rat IL after retrieval of either a fear or CTA memory leads to lasting reductions in fear and aversion responses during extinction. These findings are novel as they provide a new tool to ameliorate extinction impairments of aversive memories, and could have implications for understanding mPFC dysfunction in neuropsychiatric disorders characterized by pervasive learned fear, such as PTSD and phobias, and possibly open up novel therapeutic options for these disorders.
